# Effectiveness of sofosbuvir-based treatments for patients with hepatitis C virus genotype 6 infection: a real-world study from East China

**DOI:** 10.3389/fmed.2024.1462706

**Published:** 2024-11-26

**Authors:** Fan-Rong Jiang, Xiao-Ting Ye, He-Qing Huang, Yu-Tao Hu, Dong-Hui Wang, Su-Wen Jiang, Jia-Lan Wang, Ai-Rong Hu

**Affiliations:** ^1^Department of Pharmacy, Ningbo No. 2 Hospital, Ningbo, China; ^2^Department of Infectious Diseases, Ruian People’s Hospital, Ruian, China; ^3^Department of Infectious Diseases, Zhuji People’s Hospital, Zhuji, China; ^4^Department of Infectious Diseases, Xiangshan Hospital Affiliated to Wenzhou Medical University, Ningbo, China; ^5^Liver Diseases Center, Ningbo No. 2 Hospital, Ningbo, China; ^6^Cixi Biomedical Research Institute, Wenzhou Medical University, Wenzhou, China

**Keywords:** hepatitis C virus, genotype 6, sofosbuvir-based treatments, effectiveness, chronic hepatitis C

## Abstract

**Background:**

Over the past decade, the proportion of hepatitis C virus (HCV) genotypes (GT) 1 and 2 has decreased in almost all regions of China, while GT 3 and 6 have emerged as new challenges. GT 6 is unique in many respects, like high genetic variability and emerging resistant variants. This study aims to assess the efficacy of sofosbuvir (SOF)-based treatments in patients with GT 6 chronic hepatitis C (CHC).

**Methods:**

A retrospective analysis was conducted on patients with GT 6 HCV infection, who were diagnosed between July 2018 and May 2023. All patients received a 12-week course of SOF-based treatments. The primary efficacy endpoint was sustained virologic response (SVR), which is defined as having undetectable HCV RNA at 12 weeks after treatment completion (SVR12). The efficacy data for SVR12 were analyzed using both the evaluated population (EP) and per-protocol population (PP). For the PP populations, efficacy data were stratified using Forrester plots.

**Results:**

A total of 201 patients were included in the study. In PP population, the end of treatment virological response rate was 99.48% (190/191), the SVR12 rate was 99.31% (143/144), and the SVR24 rate was 100.00% (75/75). Only one patient with genotype 6a experienced a relapse 12 weeks after the completion of treatment, but her HCV RNA was undetectable both at the end of treatment and 24 weeks after the end of treatment. Additionally, the normalization rates of alanine transaminase (ALT) and aspartate aminotransferase (AST) were significantly higher at the end of treatment (EOT) compared to baseline (27.36% vs. 93.03%, 36.32% vs. 95.02%, *p* < 0.001). Significant improvements were observed in the levels of total bilirubin, ALT, AST, albumin, globulin, albumin/globulin ratio, gamma-glutamyl transferase, alkaline phosphatase, platelet, fibrosis-4 (FIB-4), and aspartate transaminase to platelet ratio index (APRI) between baseline and EOT (*p* < 0.05).

**Conclusion:**

SOF-based treatments achieved high virological and biochemical response rates in patients with HCV GT 6 infection.

## Introduction

Hepatitis C virus (HCV) infection is a significant cause of chronic liver disease, cirrhosis, hepatocellular carcinoma (HCC), and liver-related mortality on a global scale, imposing substantial health and economic burdens (1, 2). Notably, chronic hepatitis C (CHC) ranks among the primary causes of liver cancer deaths for both females and males. In 2020, the estimated global prevalence of HCV reached 58.5 million, with over 350,000 HCV-related deaths occurring annually (1). The rising incidence and mortality rate of HCC worldwide present substantial challenges to achieving the ambitious goal of global elimination by 2030 (3, 4).

The therapeutic landscape for CHC has undergone a remarkable evolution with the introduction of oral direct-acting antiviral agents (DAAs). Data indicate that these all-oral DAAs not only significantly reduce the risk of incident HCC and cardiovascular events but also achieve sustained virological response (SVR) rates exceeding 95% in patients (5). These therapeutic agents offer shorter treatment durations, a favorable safety profile, excellent tolerability, and documented clinical benefits, including improved liver function and substantial reductions in overall and liver-related mortality (6–9).

One such DAA combination is sofosbuvir/velpatasvir (SOF/VEL), which combines the nonstructural protein 5A inhibitor velpatasvir with the nucleotide polymerase inhibitor sofosbuvir. SOF/VEL stands as the first approved all-oral, single-tablet, pan genotypic regimen, demonstrating high efficacy against genotypes 1–6, mixed infections, and cases with unknown genotypes (10, 11). Other pan-genotypic regimens based on SOF include SOF/Ledipasvir (SOF/LDV) and SOF/Velpatasvir/Voxilaprevir (SOF/VEL/VOX).

HCV has been found to have eight primary genotypes (GT) and multiple subtypes. Each GTs exhibits distinct characteristics and is linked to the prognosis and the response to antiviral therapy. Compared to other GTs, GT6 is the most genetically diverse, classified into multiple subtypes, and has the greatest genetic variability and complexity among subtypes (12–14). Furthermore, Cirrhosis patients with GT6 are at a higher risk of developing hepatocellular carcinoma (HCC) (15). The currently available SOF-based DAA regimen has achieved ideal sustained virologic response at 12 weeks post-treatment (SVR12) in all Chinese GT6a HCV-infected patients, with good tolerability and safety. However, these results need to be confirmed in a larger population, and there are fewer studies on the efficacy of S0F-based DAA regimens in patients with GT6 HCV infection (16–18).

In China, HCV GTs comprise four types (1–3, 6, and), with GT 1 being predominant (49.2%), followed by genotypes 2, 3, and 6, accounting for 22.4, 16.4, and 11.9%, respectively (14, 19). Studies have demonstrated that the top five subtypes of HCV infection are 1b, 2a, 3b, 6a, and 3a, and the proportion of genotype 6 has been on the rise over the years, particularly in southern regions of mainland China (20, 21). Infections with GT 6 are mainly observed among people in the age group of 30 to 50. It is predominantly transmitted through intravenous drug use (14). Therefore, to achieve the 2030 Action Plan for the Elimination of Viral Hepatitis, attention must be paid to HCV genotype 6 infection in these populations (22). Nevertheless, studies focusing on the efficacy of DAAs in patients with HCV GT 6 remain limited, with most existing research primarily examining GT 1 and 2 (11, 23). Therefore, our retrospective study aimed to assess the effectiveness of SOF-based treatments in patients with HCV GT 6 infection.

## Methods

### Data collection

From July 2018 and May 2023, 201 consecutive patients aged 18 years and above with GT 6 chronic HCV infection were included in this study conducted in four hospitals. The patients or outpatient visits were recruited from the Liver Diseases Center, Ningbo No. 2 Hospital, the Department of Infectious Diseases, Ruian People’s Hospital, the Department of Infectious Diseases, Zhuji People’s Hospital, and the Department of Infectious Diseases, Xiangshan Hospital Affiliated to Wenzhou Medical University, Zhejiang Province, China. These patients received a 12-week course of treatment with SOF-based treatments. Patients with non-cirrhotic or compensatory cirrhosis who failed DAA treatment had SOF/VEL/VOX used as a rescue program (24). Patients with decompensated cirrhosis also received ribavirin as part of their treatment. Treatment regimens followed the guidance or guideline provided by the American Association for the Study of Liver Diseases-Infectious Diseases Society of America (2023), the Chinese Society of Hepatology, and Chinese Society of Infectious Diseases (2022), as well as the recommendations of the European Association for the Study of the Liver (2020) (25–27).

Patients who met any of the following criteria were excluded: age below 18, autoimmune hepatitis, primary biliary cholangitis or primary sclerosing cholangitis, genetic liver diseases, active or a history of HCC and pregnancy. Additionally, we included two groups: the evaluated population (EP) and per-protocol population (PP). Patients with no follow-up or poor adherence and under-resourced patients were included in the assessment population (EP).

All patients underwent a comprehensive clinical examination, and HCV RNA levels were monitored at baseline, and at 12, 24, and 36 weeks after treatment initiation. The following parameters were recorded at baseline and 12 weeks after treatment initiation: total bilirubin (TBil), albumin (ALB), globulin (GLB), albumin/globulin ratio (A/G), alanine transaminase (ALT), aspartate aminotransferase (AST), gamma-glutamyl transferase (GGT), alkaline phosphatase (ALP), blood platelet count (PLT), and noninvasive models such as fibrosis-4 score (FIB-4) (28) and aspartate transaminase to platelet ratio index (APRI) (29). In addition, cirrhosis and steatotic liver disease (SLD) can be diagnosed through ultrasound examination and clinical manifestations, and with one of the other imaging tests such as CT, MRI, or LSM. Hepatitis B Virus (HBV) coinfection was defined as a positive serologic hepatitis B surface antigen (HBsAg) test, evaluated with HBV DNA and HBV serologic markers (hepatitis B surface antibody, hepatitis B e antigen and hepatitis B e antibody, hepatitis B core antibody) (30). Human Immunodeficiency Virus (HIV) coinfection defined as positive HIV antigen/antibody testing and assessed by HIV RNA (31).

This study was approved by the ethics committee of Ningbo No. 2 Hospital (YJ-NBEY-KY-2022-067-01). In this study, medical data were obtained from previous clinical diagnosis and treatment, and informed consent was exempted.

### Blood test

Complete blood count was detected using Sysmex XN-1000 automated hematology analyzer (Sysmex Corporation, Japan). Serum liver function was detected with Simens Advia Chemistry XPT system analyzer (Siemens Healthcare, Germany). Serum HCV RNA was measured by real-time fluorescence quantitative PCR (ABI 7500, Applied Biosystems, CA, USA) and HCV nucleic acid quantitative detection kit (Sansure Biotech Inc. Changsha, Hunan Province, China) with the lowest detection value of 25 IU/ml (superparamagnetic nano bead method). Serum HCV genotype detection was measured by direct sequencing method (ABI 3130 gene sequencer, Applied Biosystems, USA) and the corresponding detection kit was provided by Thermo Fisher Scientific Life Technologies Corporation (Guangzhou Life Technologies, China). Same quality control standards were employed.

### Assessment of effectiveness

The primary objective of this study was to evaluate the sustained viral response (SVR12), which was defined as the absence of detectable HCV RNA (< 25 IU/ml) at 12 weeks after the completion of treatment. And SVR12 was evaluated in EP and PP populations. Additional virological response assessments included the end of treatment virological response (ETVR) and 24 weeks of off-therapy follow-ups (SVR24). A relapse was defined as the reappearance of HCV RNA after its previous absence during or after therapy. In addition, clinical biochemical markers at baseline and the end of treatment (EOT), as well as noninvasive models such as FIB-4 and APRI were assessed.

### Statistical analysis

Categorical data, whether nominal or ordinal, were presented as percentages, non-normally distributed measurement data were presented as medians with the 25th and 75th percentiles [M (*P*25, *P*75)]. Wilcoxon signed rank test for non-parametric test was used to compare data before and after treatment, and the chi-square test was employed for comparisons between two groups. SVR 12 rates (primary endpoint) and bilateral 95% exact confidence intervals (*CI*) were calculated using binomial distribution (Clopper-Pearson method). Genotypes and subgroups were analyzed using the same methods. All statistical tests were two-tailed, and significance was defined as *p* value <0.05. Statistical analyses were conducted using SPSS Statistics version 26.0 (SPSS Inc., IL, USA) and R (R packages “forestplot”).

## Results

### Enrolled patients

**Figure 1:**
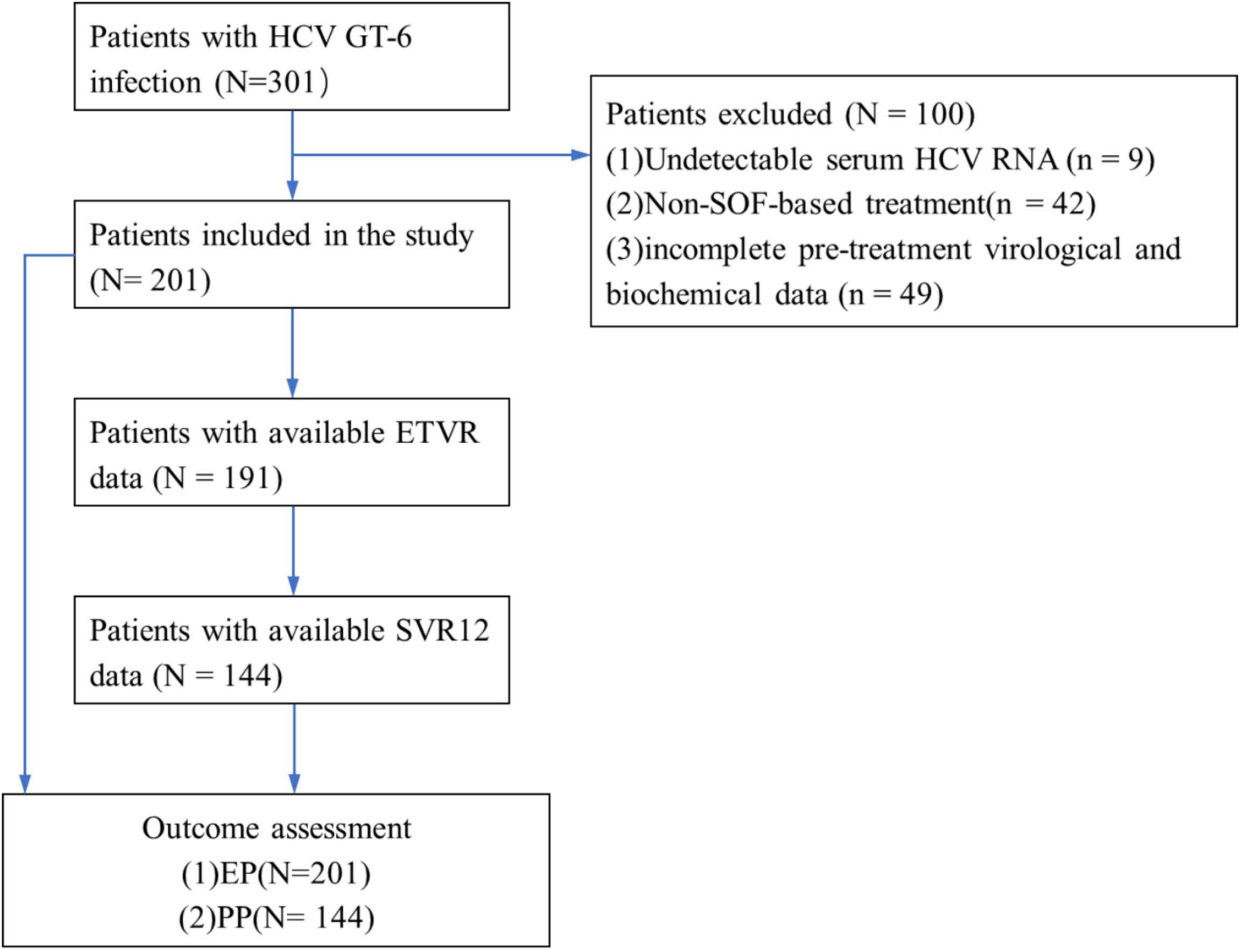
Flow diagram of enrolled patients. HCV, hepatitis C virus; GT, genotype; SVR12, the sustained viral response; ETVR, end of treatment virological response.

From July 2018 to May 2023, a total of 301 patients were diagnosed with HCV GT 6 infection. Of these, 100 patients were excluded: 9 patients had HCV RNA-negative results, 42 cases received treatment without SOF/VEL, SOF/LDV, or SOF/VEL/VOX, and 49 cases had incomplete liver function data among 3 patients received ribavirin in addition to SOF/LDV. Finally, 201 patients were included in this study, with 177 (88.06%) having GT 6a and 24 (11.94%) having GT 6n and all received treatment with SOF/VEL. Following SOF-based treatment, 57, 10, 57, and 126 patients had undetectable HCV RNA at 12, 24, and 36 weeks after treatment initiation, respectively. In addition, 201 patients were included the evaluation population (EP) and 144 patients were included in per-protocol population (PP). The flow diagram of the study population is shown in Figure 1.

### Baseline characteristics

The mean age of the patients was 48.84 ± 12.76 years, with 133 (66.17%) being male and the median HCV RNA value was 6.46 (5.58–6.92) Log IU/ml. Liver cirrhosis was present in 12 patients (5.97%), 23 patients (11.44%) had HBV coinfection and all patients underwent antiviral HBV treatment, 53 patients (26.37%) had SLD, 3 patients (1.49%) were co-infected with HIV and all on anti-HIV treatment (Table 1). Table 1 also provides information on the transmission routes of the patients, with the majority (143, 71.14%) having an unclear route of infection. Among the remaining patients, 14 (6.97) had the parenteral transmission, 36 (17.91%) had taking drugs, some (8, 3.98%) had Mother-to-child transmission.

**Table 1:** Patient baseline characteristics.

Characteristics	Sofosbuvir-based treatments cohort (*n* = 201)
Male, *n* (%)	133 (66.17)
Age (years)	48.33 (42.00–55.17)
GT-6 subtype, *n* (%)	
6a	177 (88.06)
6n	24 (11.94)
FIB-4 > 3.25 (%)	43 (21.39)
Cirrhosis, *n* (%)	12(5.97)
HBV coinfection, *n* (%)	23 (11.44)
SLD, *n* (%)	53 (26.37)
HIV coinfection, *n* (%)	3 (1.49)
Route of infection, *n* (%)	
Mother-to-child transmission	8 (3.98)
Taking drugs	36 (17.91)
The parenteral transmission	14 (6.97)
Unknown	143 (71.14)
Treatments, *n* (%)	
SOF/VEL	180 (89.55)
SOF/LDV	19 (9.45)
SOF/VEL/VOX	2 (1.00)
Other treatments, *n* (%)	
HBV treatment	23 (11.44)
HIV treatment	3 (1.49)
Blood test	
HCV RNA, median (Q1–Q3), Log IU/ml	6.46 (5.58–6.92)
TBil, median (Q1–Q3), μmol/L	14.50 (10.90–10.60)
ALB, median (Q1–Q3), g/L	43.00 (39.50–45.00)
GLB, median (Q1–Q3), g/L	30.00 (26.90–33.70)
A/G, median (Q1–Q3),	1.44 (1.25–1.63)
ALT, median (Q1–Q3), U/L	71.00 (34.50–153.50)
AST, median (Q1–Q3), U/L	51.00 (29.00–103.50)
GGT, median (Q1–Q3), U/L	57.00 (26.00–121.00)
ALP, median (Q1–Q3), U/L	89.00 (68.00–118.50)
PLT, median (Q1–Q3), ×10 (9)/L	191.00 (150.00–217.00)
FIB-4, median (Q1–Q3)	1.68 (1.02–2.71)
APRI, median (Q1–Q3)	0.74 (0.38–1.62)

GT, genotype; HCV, hepatitis C virus; HBV: hepatitis B virus infection; SLD: steatotic liver disease; HIV: human immunodeficiency virus infection; SOF/VEL, Sofosbuvir/Velpatasvir; SOF/LDV, Sofosbuvir/Ledipasvir; SOF/VEL/VOX, Sofosbuvir/Velpatasvir/ Voxilaprevir. HBV, hepatitis B virus; GT, genotype; TBil, total bilirubin; ALB, albumin; GLB, globulin; A/G, albumin/globulin; ALT, alanine transaminase; AST, aspartate aminotransferase; GGT, gamma-glutamyl transferase; ALP, alkaline phosphatase; PLT, blood platelet; FIB-4, fibrosis-4, FIB-4 = [age (years) × AST (U/L)]/{platelet count [10 (9)/L] × [ALT (U/L)^1/2^]} (28); APRI, aspartate transaminase to platelet ratio index, APRI = {(AST/ULN)/platelet counts [10 (9)/L]} × 100 (29); HCV, hepatitis C virus. Quantitative data of normal distribution were expressed as mean ± standard deviation, non-normal distribution data were expressed as median (Q1-Q3), and categorical data were expressed as frequency and percentage. The diagnostic criteria for cirrhosis: through ultrasound examination and clinical manifestations, and with one of the other imaging tests such as CT, MRI, or LSM.

Compared to mono-HCV infection, CHC patients with HBV co-infection had lower ALT and PLT levels (*p* = 0.017, 0.033), while the median age of CHC patients with SLD appeared to be lower but not statistically significant (49.79 *VS* 46.21, *p* = 0.080). Compared to GT 6n, patients with HCV GT 6a infection had a higher proportion of males (*p* = 0.074), lower age (*p* < 0.001) and lower FIB-4 levels (*p* = 0.010).

### Treatment effectiveness

#### Virologic response

**Figure 2:**
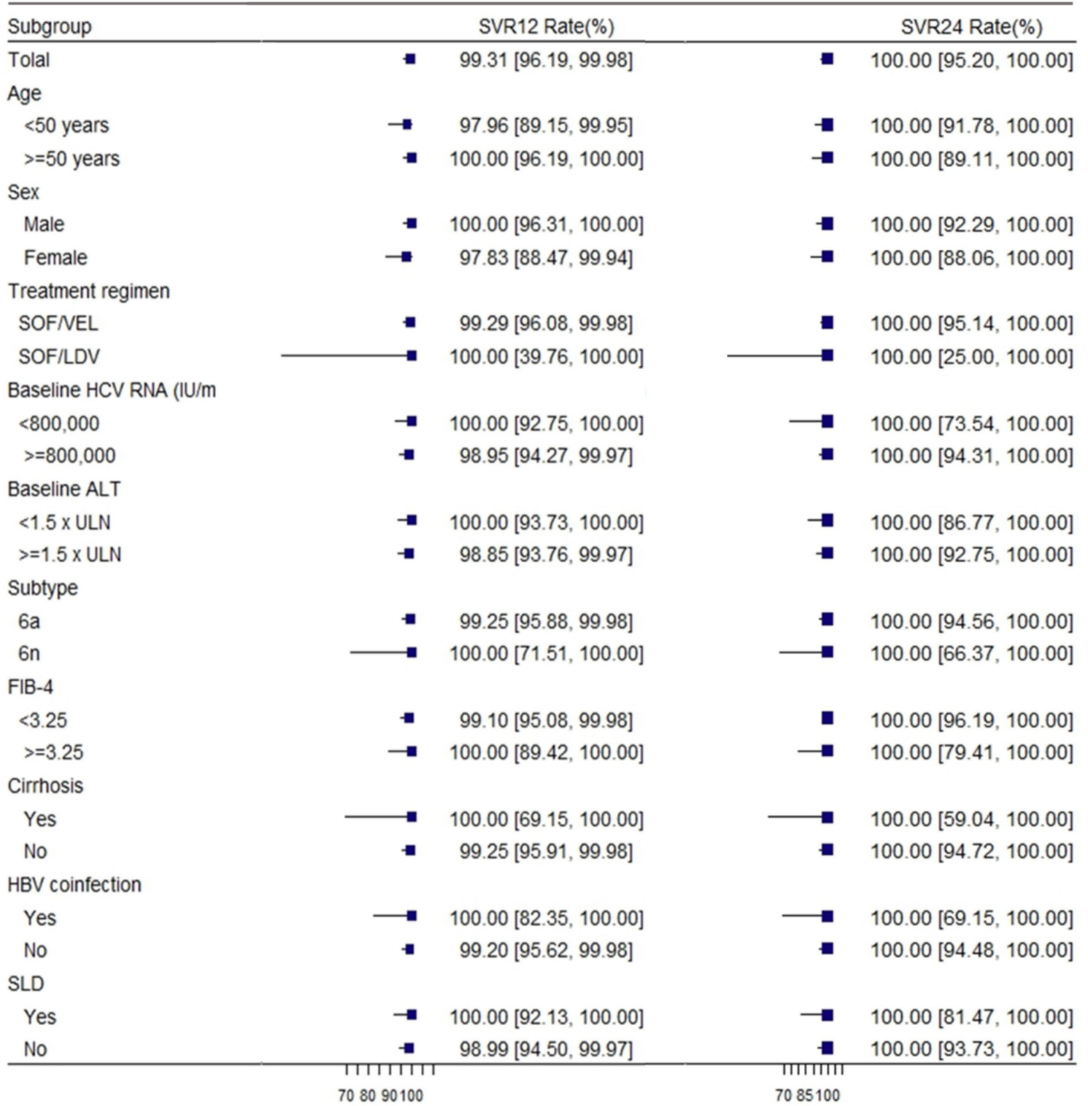
Rates of sustained virologic response according to subgroup. SVR12, sustained virological response at 12 weeks; SVR24, sustained virological response at 24 weeks; HCV, hepatitis C virus; GT, genotype; SLD: steatotic liver disease. FIB-4 = [age (years) × AST (U/L)]/{platelet count [10 (9)/L] × [ALT (U/L)^1/2^]} (28).

In our study, we reported the proportion of patients achieving ETVR, SVR12, and SVR24 using EP and PP. In the EP population, 190 of 201 patients (94.53%; 95% *CI:* 90.42–97.24%) achieved ETVR, 143 achieved SVR 12 (71.13, 95% *CI:* 64.35–77.32%), and 75 (37.31, 95% *CI:* 30.61–44.40%) achieved SVR 24.ETVR was 99.48% (190/191, 95% *CI:* 97.11–99.99%), SVR12 was 99.31% (143/144, 95% *CI:* 96.19–99.98%) and SVR24 was 100% (75/75, 95% *CI:* 95.20–100.00%) in per-protocol (PP) population (Table 2). Considering the extremely high level of sustained virologic response observed overall, high response rates were witnessed in all patient subgroups. The response rates of individual subgroups were similar to those of patients without these characteristics (as shown in Figure 2).

**Table 2:** Virologic response post-treatment.

Response	EP	PP
ETVR, *n* (%), [95% *CI*]		
Any genotype	190/201 (94.53) [90.42, 97.24]	190/191 (99.48) [97.11, 99.99]
6a	168/177 (94.92) [90.57, 97.65]	168/168 (100.00) [97.83, 100.00]
6n	22/24 (91.67) [73.00, 98.97]	22/23 (95.65) [78.05, 99.89]
SVR12, *n* (%), [95% *CI*]		
Any genotype	143/201 (71.13) [64.35, 77.32]	143/144 (99.31) [96.19, 99.98]
6a	132/177 (65.67) [58.66, 72.21]	132/133 (99.25) [95.88, 99.98]
6n	11/24 (45.83) [25.56, 67.18]	11/11 (100.00) [71.51, 100.00]
SVR24, *n* (%), [95% *CI*]		
Any genotype	75/201 (37.31) [30.61, 44.40]	75/75 (100.00) [95.20, 100.00]
6a	66/177 (37.29) [30.15, 44.86]	132/133 (99.25) [95.88, 99.98]
6n	9/24 (37.50) [18.80, 59.41]	11/11 (100.00) [71.51, 100.00]

All values are n (%) unless stated otherwise. EP: evaluated population; PP: per-protocol population; ETVR: the end of treatment virological response; SVR12: sustained viral response at 12 weeks; SVR24: sustained viral response at 12 weeks.

One of the patients with genotype 6a had a relapse 12 weeks after the end of treatment. This patient was a middle-aged female, with a median RNA value of 934000 IU/ml. However, her HCV RNA was undetectable at the end of treatment and after EOT of 24 weeks. Among patients with different genotypes, there was no discernible variation in the virologic response rate at different visit periods. Regardless of the patient’s baseline status, the values of SVR12 were close to or exceeded 99%, and the values of SVR24 were 100%.

#### Changes in laboratory index and liver fibrosis

Comparing the EOT with the pre-treatment period, significant decreases were observed in TBil, ALB, GLB, A/G, ALT, AST, GGT, ALP, PLT, FIB-4 and APRI (all *p* < 0.05, Table 3). The ALT normalization rate at EOT was 93.03% (187/201), significantly higher than that at baseline (93.03% vs. 27.36%, *p* < 0.001). The AST normalization rate at EOT was 95.02% (191/201), also significantly higher than that at baseline (95.02% vs. 36.32%, *p* < 0.001). At EOT, compared to GT 6n (95.83%), patients with GT 6a (82.49%) showed a lower rate of ALT normalization and patients with baseline cirrhosis had a lower rate of AST normalization (83.33%) than those without cirrhosis (95.77%). Additionally, patients with baseline liver cirrhosis showed a higher TBil, ALT, AST, GGT, FIB-4, and APRI levels, and a lower PLT level. Patients with HBV co-infection showed higher ALP, FIB-4, and APRI levels, and lower levels of GLB and PLT, while patients complicated with SLD showed a higher GGT level than patients without such conditions (all *p* < 0.05, Supplementary Table).

**Table 3:** Distribution of characteristics in 201 patients at baseline and end of treatment.

Parameters	Baseline	EOT	*Z*/*t* value	*p* value
TBil, median (Q1–Q3), μmol/L	14.50 (10.90–20.60)	11.50 (8.90–14.85)	5.462	< 0.001
ALB median (Q1–Q3), g/L	43.00 (39.50–45.00)	43.90 (42.00–45.40)	−3.862	< 0.001
GLB, median (Q1–Q3), g/L	30.00 (26.90–33.70)	28.80 (26.10–31.60)	2.508	0.013
A/G, median (Q1–Q3),	1.44 (1.25–1.63)	1.53 (1.35–1.70)	−2.844	0.005
ALT, median (Q1–Q3), U/L	71.00 (34.50–153.50)	16.00 (12.00–23.00)	14.263	< 0.001
AST, median (Q1–Q3), U/L	51.00 (29.00–103.50)	21.00 (17.00–24.00)	13.429	< 0.001
GGT, median (Q1–Q3), U/L	57.00 (26.00–121.00)	24.00 (17.00–43.50)	7.840	< 0.001
ALP, median (Q1–Q3), U/L	89.00 (68.00–118.50)	75.00 (62.00–94.00)	4.224	< 0.001
PLT, median (Q1–Q3), ×10 (9)/L	191.00 (150.00–217.00)	201.00 (163.00–235.00)	−2.164	0.030
FIB-4, median (Q1–Q3)	1.68 (1.02–2.71)	1.22 (0.84–1.92)	4.521	< 0.001
APRI, median (Q1–Q3)	0.74 (0.38–1.62)	0.25 (0.19–0.35)	11.908	< 0.001

EOT, end of treatment; TBil, total bilirubin; ALB, albumin; GLB, globulin; A/G, albumin/globulin; ALT, alanine transaminase; AST, aspartate aminotransferase; GGT, gamma-glutamyl transferase; ALP, alkaline phosphatase; PLT, blood platelet; FIB-4, fibrosis-4, FIB-4 = [age (years) × AST (U/L)]/{platelet count [10 (9)/L] × [ALT (U/L)^1/2^]} (28); APRI, aspartate transaminase to platelet ratio index, APRI = {(AST/ULN)/platelet counts [10 (9)/L]} × 100 (29).

## Discussion

Our study is a retrospective real-world analysis that examined the efficacy of SOF based treatment in patients with GT 6 HCV from July 2018 to May 2023. We carried out a follow-up period of 36 weeks, is longer than that in previous studies, enabling us to more accurately assess the effectiveness of the drug. In addition, our study analyzed subgroups of patients based on their baseline data to evaluate the efficacy of SOF-based therapy in HCV GT6-infected patients. The patient population in this study consisted of individuals from various regions in East China. Among the 201 patients with GT 6 HCV infection, the majority were relatively young, and there was a high proportion of drug-addicted individuals, which aligns with previous findings in the Wenzhou region of East China (32). This underscores the potential role of drug addiction in CHC transmission, particularly among GT 6 patients in this region. Therefore, it is crucial to implement better control measures targeting the drug-using population to prevent further spread of HCV infection.

The efficacy of SOF-based combinations in treating GT 6 chronic hepatitis C was remarkable, with an SVR12 rate of 99.31% and SVR24 at 100%. These results are comparable to or even higher than the efficacy observed in other GTs, except for GT 1 and GT 2 (33). Furthermore, we reported ETVR, SVR12, and SVR24 using the EP and PP populations. A high degree of concordance in ETVR, SVR12, and SVR24 could be found in the PP population, and subgroup analyses of SVR12 according to age, gender, treatment regimen, HCV RNA, subtype, FIB-4, ALT, HBV co-infection, and steatotic liver disease (SLD) revealed that a high response rate was observed in Similar high response rates were observed in all patient subgroups. These factors had little effect on SVR12. However, in the EP population, there is a high degree of variability in ETVR, SVR12, and SVR24. In real-world clinical practice, these two populations may not always achieve the same efficacy and safety. In addition, subgroup analyses demonstrated that the SOF-based regimen achieved a 100% sustained virological response at 12 weeks among patients with cirrhosis, steatohepatitis, and those co - infected with HBV. For both genotypes 6a and 6n, the SVR12 rate was more than 99%. The patient who experienced relapse in our study was a 50-year-old woman with none underlying disease. Her baseline HCV RNA level was moderate at 934000 IU/ml, but at the 12-week follow-up after EOT, her HCV RNA level rose to 199,000 IU/ml. Nevertheless, EOT and 24-week follow-up, her HCV RNA was undetectable. Reinfection and irregular premedication use may have contributed to her relapse.

We also investigated the changes in laboratory indices and liver fibrosis markers before and after SOF-based treatment. Significant improvements were observed in liver function parameters such as TBil, ALB, GLB, A/G, AST, ALT, GGT, ALP, PLT, FIB-4 and APRI from baseline to the end of treatment, consistent with previous findings (34). High rates of ALT and AST normalization were noted at the end of treatment. A significant difference in the effect of genotype on ALT at 4 weeks of treatment, with GT 6n being more effective than GT 6a. Given the fact that liver cirrhosis is a serious disease, patients with liver cirrhosis had a higher effect on liver function than other conditions, with lower PLT level as well as higher FIB-4 and APRI levels. In addition, our study revealed that patients with baseline liver cirrhosis had a considerably lower incidence of AST normalization at 4 weeks of treatment compared to other groups. The condition of CHC complicated with HBV infection was relatively more severe than other conditions. Also, these patients had lower PLT level, and may receive anti-HBV therapy with lower ALT. As a result, adjuvant therapy and close follow-up is necessary for these patients.

Despite these positive outcomes, our study has some limitations. Firstly, the number of GT 6n patients included was relatively small, and there were some patients lost to follow-up at each stage. Further investigation is needed to determine the reasons behind these instances of relapse and loss to follow-up. Secondly, due to the insidious nature of HCV infection, most patients found it difficult to determine the transmission routes or were unwilling to disclose it. Only 61 (30.35%) cases were able to obtain the route of infection in this study. Thirdly, our study did not include data analysis specifically for pediatric patients under 18 years of age, despite previous research demonstrating the efficacy and safety of SOF-based regimens in treating CHC in this age group (11). Moreover, two available commercial HCV genotyping assays, namely the Abbott and Roche systems, are employed in clinical practice. The diagnostic accuracy of these assays in detecting major HCV genotypes 1–6 and HCV GT-1 subtypes 1a and 1b exceeds 95%. However, the performance of these assays in detecting HCV GT-6, especially not HCV GT-6a, tends to be suboptimal (35).

In conclusion, our real-world cohort study underscores the effectiveness of SOF-based treatment in achieving high virological and biochemical responses in patients with GT 6 chronic hepatitis C. Continued research is warranted to address these limitations and to further refine treatment strategies.

## Data Availability

Data are available from the author upon reasonable request by sending an email to huairong@ucas.edu.cn.
