# Supplementary material for: Effectiveness of sofosbuvir-based treatments for patients with hepatitis C virus genotype 6 infection: a real-world study from East China
Source: Front Med (Lausanne). 2024 Nov 26;11:1462706. doi: 10.3389/fmed.2024.1462706 (PMC11628284; doi:10.3389/fmed.2024.1462706)
Supplement: Supplementary file 1 [file Table_1.DOC]

Supplementary Material

**Supplement Table. Characteristics in 201 patients at end of treatment in different groups**

| **Parameters** | **Liver cirrhosis** | | **Complicated with HBV infection** | | **Complicated with fatty liver disease** | | **GT** | | ***Z*/*t* value** | ***P* value** |
| --- | --- | --- | --- | --- | --- | --- | --- | --- | --- | --- |
|  | No (*N* = 189) | Yes (*N* = 12) | No (*N* = 178) | Yes (*N* = 23) | No (*N* = 148) | Yes (*N* = 53) | 6a (*N* = 177) | 6n (*N* = 24) |  |  |
| TBil, median (Q1–Q3), μmol/L | 11.40  (8.75–14.60) | 16.70  (11.97-22.70) | 11.55  (8.77-14.92) | 11.00 (9.70-14.20) | 11.60  (8.62-15.20) | 10.90 (9.20-14.55) | 11.40 (8.85–14.75) | 12.45 (9.27–17.20) | -2.608, -0.318, -0.204, -0.862 | 0.009, 0.750, 0.839, 0.389 |
| ALB, mean ± SD, g/L | 43.72 ± 3.22 | 43.89 ± 3.37 | 43.74 ± 3.20 | 43.61 ± 3.47 | 43.69 ± 2.78 | 43.84 ± 4.24 | 43.71 ± 3.27 | 43.89 ± 2.89 | -0.181, 0.188, -0.294, -0.265 | 0.856, 0.851, 0.769, 0.791 |
| GLB, mean ± SD, g/L | 29.04 ±4.92 | 31.22 ± 6.23 | 29.42 ± 4.75 | 27.18 ± 6.52 | 29.36 ± 5.30 | 28.62 ± 4.11 | 29.10 ± 5.06 | 29.62 ± 4.78 | -1.470, 2.031, 0.923, -0.472 | 0.143, 0.044, 0.357, 0.638 |
| A/G, mean ± SD | 1.54 ± 0.27 | 1.44 ± 0.23 | 1.52 ± 0.27 | 1.58 ± 0.24 | 1.53 ± 0.27 | 1.54 ± 0.24 | 1.53 ± 0.27 | 1.52 ± 0.26 | 1.183, -0.966, -0.265, 0.168 | 0.238, 0.335, 0.791, 0.867 |
| ALT, median (Q1–Q3), U/L | 15.00 (11.50-23.00) | 23.50 (15.25-31.25) | 15.00 (11.75-23.00) | 22.00 (12.00-26.00) | 15.00 (11.00-22.00) | 18.00 (13.00-26.00) | 16.00 (12.00–23.20) | 14.00 (11.00–17.75) | -2.167, -1.573, 1.592, -1.735 | 0.030, 0.116, 0.051, 0.083 |
| AST, median (Q1–Q3), U/L | 20.00 (17.00-23.00) | 26.00 (20.50-36.75) | 20.00 (17.00-23.00) | 24.00 (19.00-29.00) | 20.00 (17.00-23.00) | 21.00 (18.00-26.00) | 20.00 (17.00–24.00) | 22.50 (18.25–24.75) | -2.429, -2.737, -1.298, -0.768 | 0.015, 0.006, 0.194, 0.442 |
| GGT, median (Q1–Q3), U/L | 23.00 (16.00-40.50) | 44.50 (34.00-61.50) | 24.00 (16.75-122.25) | 24.00 (17.00-49.00) | 23.00 (16.00-41.25) | 29.00 (20.00-53.00) | 25.00 (17.00–44.00) | 23.00 (16.00–37.25) | -2.652, -0.798, -2.270, 0.795 | 0.008, 0.425, 0.023, 0.427 |
| ALP, median (Q1–Q3), U/L | 75.00 (62.00-93.00) | 89.00 (64.75-116.25) | 74.50 (62.00-92.00) | 92.00 (66.00-105.00) | 73.00 (62.00-93.75) | 83.00 (65.50-94.50) | 75.00 (62.40–93.50) | 73.50 (62.00–98.75) | -1.408, -2.103, -1.269, 0.017 | 0.159, 0.035, 0.204, 0.987 |
| PLT, median (Q1–Q3), ×10^9^/L | 203.00 (168.00-237.00) | 148.00 (88.50-180.00) | 208.50 (167.75-237.25) | 176.00 (144.00-202.00) | 201.00 (165.25-237.75) | 201.00 (145.50-231.50) | 201.00 (162.00–232.00) | 197.50 (168.00–251.25) | 3.524, 2.716, 0.516, 0.202 | < 0.001, 0.007, 0.606, 0.840 |
| FIB-4, median (Q1–Q3) | 1.21  (0.84-1.84) | 2.17  (1.26-3.79) | 1.19  (0.82-1.85) | 1.47  (1.18-2.37) | 1.24  (0.84-1.91) | 1.19  (0.84-2.01) | 1.18  (0.82–1.72) | 1.89  (1.38–2.31) | -2.766, -2.537, 0.288, -3.360 | 0.006, 0.011, 0.774, 0.001 |
| APRI, median (Q1–Q3) | 0.25  (0.19-0.34) | 0.52  (0.32-0.76) | 0.24  (0.18-0.34) | 0.33  (0.28-0.48) | 0.25  (0.18-0.33) | 0.27  (0.20-0.41) | 0.25  (0.19–0.35) | 0.28  (0.20–0.38) | -3.275, -3.524, -1.275, -0.406 | 0.001, < 0.001, 0.202, 0.685 |

***Abbreviations:*** HBV, hepatitis B virus; GT, genotype; TBil, total bilirubin; ALB, albumin; GLB, globulin; A/G, albumin/globulin; ALT, alanine transaminase; AST, aspartate aminotransferase; GGT, gamma-glutamyl transferase; ALP, alkaline phosphatase; PLT, blood platelet; FIB-4, fibrosis-4, FIB-4=(age (years)×AST (U/L))/(platelet count (10^9^/L)×[ALT (U/L)^1/2^]) ^1^; APRI, aspartate transaminase to platelet ratio index, APRI=[(AST/ULN)/platelet counts (10^9^/L)]×100 ^2^.

Quantitative data of normal distribution were expressed as mean ± standard deviation, non-normal distribution data were expressed as median (Q1-Q3), and categorical data were expressed as frequency and percentage.

**Reference：**

1. Sterling, R. K.; Lissen, E.; Clumeck, N.; Sola, R.; Correa, M. C.; Montaner, J.; M, S. S.; Torriani, F. J.; Dieterich, D. T.; Thomas, D. L.; Messinger, D.; Nelson, M., Development of a simple noninvasive index to predict significant fibrosis in patients with HIV/HCV coinfection. *Hepatology* **2006,** *43* (6), 1317-25.

2. Lin, Z. H.; Xin, Y. N.; Dong, Q. J.; Wang, Q.; Jiang, X. J.; Zhan, S. H.; Sun, Y.; Xuan, S. Y., Performance of the aspartate aminotransferase-to-platelet ratio index for the staging of hepatitis C-related fibrosis: an updated meta-analysis. *Hepatology* **2011,** *53* (3), 726-36.
